# A phenomenology of meditation-induced light experiences: traditional buddhist and neurobiological perspectives

**DOI:** 10.3389/fpsyg.2013.00973

**Published:** 2014-01-03

**Authors:** Jared R. Lindahl, Christopher T. Kaplan, Evan M. Winget, Willoughby B. Britton

**Affiliations:** ^1^Department of Religious Studies, Warren Wilson CollegeAsheville, NC, USA; ^2^Department of Psychiatry and Human Behavior, Warren Alpert Medical School at Brown UniversityProvidence, RI, USA

**Keywords:** meditation, buddhism, concentration, light, hallucinations, neuroplasticity

## Abstract

The scientific study of Buddhist meditation has proceeded without much attention to Buddhist literature that details the range of psychological and physiological changes thought to occur during meditation. This paper presents reports of various meditation-induced light experiences derived from American Buddhist practitioners. The reports of light experiences are classified into two main types: discrete lightforms and patterned or diffuse lights. Similar phenomena are well documented in traditional Buddhist texts but are virtually undocumented in scientific literature on meditation. Within Buddhist traditions, these phenomena are attributed a range of interpretations. However, because it is insufficient and problematic to rely solely upon the textual sources as a means of investigating the cause or significance of these phenomena, these qualitative reports are also considered in relation to scientific research on light-related experiences in the context of sensory deprivation, perceptual isolation, and clinical disorders of the visual system. The typologies derived from these studies also rely upon reports of experiences and closely match typologies derived from the qualitative study of contemporary practitioners and typologies found in Buddhist literary traditions. Taken together, these studies also provide evidence in support of the hypothesis that certain meditative practices – especially those that deliberately decrease social, kinesthetic, and sensory stimulation and emphasize focused attention – have perceptual and cognitive outcomes similar to sensory deprivation. Given that sensory deprivation increases neuroplasticity, meditation may also have an enhanced neuroplastic potential beyond ordinary experience-dependent changes. By providing and contextualizing these reports of meditation-induced light experiences, scientists, clinicians, and meditators gain a more informed view of the range of experiences that can be elicited by contemplative practices.

## INTRODUCTION

Meditation practices that were previously taught within the context of religious traditions are now increasingly being practiced in non-traditional and secular contexts. Since the rise of mindfulness-based interventions (MBIs) such as mindfulness-based stress reduction (MBSR), “meditation” is also being prescribed in a clinical context as treatment for a variety of psychological and physiological ailments (e.g., Kabat-Zinn et al., 1985; Kristeller and Hallett, 1999; Goldin and Gross, 2010). Current research has focused almost exclusively on the beneficial effects of meditation, such that the scientific community has not thoroughly investigated the full range of experiences that can arise as a result of meditative practices. It is important to recognize that the trajectories of practice outlined in the traditional literature of contemplative traditions include a wide range of experiences that fall outside the commonly reported positive health effects, including unusual affective, perceptual, and somatic changes (Kornfield, 1979). Especially as practitioners move into more advanced stages of practice, a number of experiences may arise that can be bewildering to those who are not expecting them and who are not prepared to manage them.

Part of the reason for this lacuna in the scientific understanding of meditation derives from the fact that scientific research on meditation, especially at the clinical level, has become increasingly divorced from the study of the literature and practitioners from contemplative traditions. Consulting traditional sources is an essential and important step in furthering the *scientific* understanding of meditation. The literature on Buddhist meditation not only clearly delineates the stages of practice that comprise contemplative disciplines, it also details a range of psychological and physiological experiences that might be expected from undertaking such practices (e.g., Nanamoli, 1997; Buddhaghosa, 1999; Dalai Lama, 2001; Namgyal, 2006; Wallace, 2011). Without adequate knowledge of the range of possible meditation-related experiences, there is a risk that in the clinical application of meditative practices – where meditation training is divorced from its traditional religious, social, and cultural contexts – reports of such experiences could be misdiagnosed as a more serious physiological or psychological disorder. In order to have realistic and accurate expectations of the possible outcomes of meditative practices, clinicians and researchers in this field should be aware of the trajectories of practice and experience detailed in traditional religious literature.

The data in this study are derived from a larger on-going project that is investigating the full range of contemplative experiences. In this paper, we focus in particular on addressing experiences described as lights or as having luminous characteristics. We have selected these experiences from among the possible range of meditation-induced experiences for three primary reasons. First, with the exception of one study that included lights among dozens of different meditation-related symptoms (Kornfield, 1979) and one study in which light-related metaphors are used to describe an experience of “inner energy” (Lo et al., 2003), lights are fairly common meditation experiences that have gone undocumented in the scientific research on meditation. Second, reports of light-related experiences are well documented in scientific literature on visual hallucinations, and their neurobiology is fairly well understood. Third, traditional Buddhist literature provides a rich typology of meditation-induced light experiences that contextualizes these experiences within the trajectories of meditative progress.

The qualitative data for this paper are derived from first-person reports of meditative experiences provided by contemporary American Buddhist practitioners in a variety of lineages. These data are contextualized in relation to typologies of meditative experiences derived from traditional Buddhist literary traditions. While Buddhist literature abounds with detailed references to experiences of lights or luminosity (e.g., Nanarama, 1983; Dondrup, 1997; Chagme and Gyatrul, 2000; Wangyal, 2000,^2002^; Gyatso, 2004; Wallace, 2011), to our knowledge, this is the first study that attempts to connect historical, textual data on meditation-induced light experiences with reports from living practitioners of different lineages as well as with related data from experimental scientific research.

Our analysis is restricted to assessing simple visual hallucinations – unstructured points of light, patterns of light, and diffuse changes in the visual field – because these are the types of experience emphasized both in the qualitative data derived from our interviews with Buddhist meditators and in the typologies of meditation-induced light experiences in Buddhist literature. However, it is insufficient to rely solely upon traditional textual sources as a means of investigating the cause or significance of these phenomena. For this reason, our qualitative data are considered in relation to scientific research on sensory deprivation (e.g., Zubek et al., 1961; Zuckerman, 1969; Merabet et al., 2004), perceptual isolation (homogenous stimuli; e.g., Wackermann et al., 2002,^2008^; Pütz et al., 2006; Lloyd et al., 2012), and clinical disorders of the visual system (e.g., Santhouse et al., 2000; Wilkinson, 2004; Vukicevic and Fitzmaurice, 2008; Ffytche et al., 2010). These studies, discussed below, also provide evidence in support of the hypothesis that certain contemplative practices – especially those that deliberately decrease social, kinesthetic, and sensory stimulation and emphasize focused attention – have perceptual and cognitive outcomes similar to sensory deprivation. Finally, we suggest that since sensory deprivation has been shown to introduce a period of enhanced neuroplasticity (Boroojerdi et al., 2000; Fierro et al., 2005; Pitskel et al., 2007; Maffei and Turrigiano, 2008), meditation may also have an enhanced neuroplastic potential beyond ordinary experience-dependent changes.

## MATERIALS AND METHODS

### PARTICIPANTS

Twenty-eight meditators (39% female, 61% male, mean age = 43.3, SD = 14.1, range = 21–74) participated in the “Varieties of Contemplative Experience” study. The subject pool was recruited via snowball sampling (Faugier and Sargeant, 1997). General inclusion criteria required a minimum age of 18 years, a regular meditation practice in one or more recognized Buddhist traditions, and a meditation-related experience that was significant, unexpected, challenging, or was associated with physiological or psychological changes. Twenty (71%) participants practiced in an American Vipassan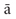 or an Asian Therav
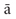
da Buddhist tradition, seven (25%) in a Tibetan Buddhist tradition, and seven (25%) in a Zen Buddhist tradition^1^. Nearly half (46%) of the sample were meditation teachers. The average number of prior years of practice before the onset of unexpected experiences was 10.33 (SD = 10.88, range = 0.25–41).

### PROCEDURE

The project was approved by the Brown University Institutional Review Board, and all participants provided informed consent. Interviews were conducted via telephone and in person either by the PI (WB) or by other study personnel (CK, EW). After preliminary demographic information was collected, subjects were interviewed in a semi-structured, open-ended format.

Since the primary data are narratives and the aim of the study is to generate descriptions of the participants’ meditation experiences, qualitative methodology is the most appropriate approach. Thus, interviews were conducted, recorded, transcribed, and coded in line with qualitative methodology standards (Miles and Huberman, 1994; Patton, 2002; Flick, 2006; Fonteyn et al., 2008; DeCuir-Gunby et al., 2011; Miles et al., 2013). Content analysis is a systematic and objective means of describing and quantifying phenomena (Krippendorff, 1980; Downe-Wamboldt, 1992; Elo and Kyngas, 2008), and may be either inductive or deductive. We chose to use inductive content analysis, where categories are derived from the data, because this is the method that is recommended if there is little former knowledge about a phenomenon (Elo and Kyngas, 2008).

Because the varieties of meditation-related experiences have not been well documented in scientific literature, it was crucial that the interview content was driven by the subject, not the researcher. Subjects were allowed to respond freely to the initial open-ended prompt “What was your experience with meditation?” Additional queries included non-specific prompts for elaboration such as “Can you tell me more about that?” In order to minimize researcher’s influence on interview content, directed queries about specific experiences or queries that interrupted the narrative were discouraged.

A total of 2157 min of audio recording were transcribed into 605 pages and separated into 6594 separate units of analysis. Within the transcripts, a new unit of analysis was created for each change in speaker, concept, or event, and each unit was allocated to a separate row in an excel spreadsheet (Cavanagh, 1997; Graneheim and Lundman, 2004; Elo and Kyngas, 2008).

The qualitative content analysis followed a grounded theory approach (Glaser and Strauss, 1967) using open coding techniques (Strauss and Corbin, 1998), which are intended to “open up” the text in order to uncover its content and meaning. Following the methodology of open coding, coders assign a tentative heading or category to each unit of analysis. They then read and code the transcript repeatedly until all aspects of the content are categorized. As the coding structure evolved from the initial open coding, the research team met repeatedly to create and revise a list of standardized definitions for each type of experience with inclusion and exclusion criteria, supported by example texts (MacQueen et al., 1998; Fonteyn et al., 2008). To establish reliability for the codebook, an interview was chosen at random and was coded independently by each of the researchers. All interviews were coded by pairs of researchers with the finalized manual, and any disagreements led to iterative discussions that refined coding criteria until consensus (80%) was achieved.

More than forty categories of experience were aggregated into six higher-order clusters: cognitive, perceptual, sense of self, affective/emotional, somatic/physiological, and social/occupational. “perceptual” is defined as pertaining to the senses, i.e. the visual, auditory, gustatory, olfactory, and tactile systems. “Light experiences” emerged as a sub-category of perceptual experiences in the visual domain. Inclusion criteria for light-related visual experiences included use of the word “light” or description of an experience either directly linked to visual perception with the phenomenal quality of luminosity or brightness. Exclusion criteria were metaphorical uses of light that were not directly linked with visual perception or that had ambiguous phenomenal quality (cf., Lo et al., 2003). Complex involuntary mental images involving objects, figures, and scenes that were not linked to a light-related experience were included in another category and therefore are not addressed in this paper. Because the purpose of this paper is to discuss light-related experiences from traditional Buddhist and neurobiological perspectives, only cells that received the <Perceptual.Visual.light> code are included below.

## RESULTS

Nine individuals (32% of total participants) voluntarily reported lights or other forms of luminous experiences [mean age = 41, SD = 9.7, range = 31–60, two females (22%), seven males (78%)]. Six (67%) of these participants practiced in an American Vipassan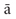 or Burmese Therav
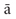
da tradition, two (22%) in a Zen Buddhist tradition, and one (11%) in a Tibetan Buddhist tradition. Five (56%) of the meditation-induced light experiences appeared on retreats, and the remaining four (44%) arose in the context of daily practice. Among these reports, the level of light of the meditation environment varied according to setting and time of day and was not intentionally manipulated. None of the practitioners engaged in a “dark retreat” practice where the level of light is deliberately attenuated. Practitioners who reported lights had been practicing within one or more Buddhist traditions for an average of 5.0 years (SD = 2.0, range = 2–8) before these experiences arose (**Table 1**).

**Table 1:** Practitioner data and reports of meditation-induced light experiences.

Subject no.	Sex	Dominant practice tradition	Duration of practice before lights (years)	Retreat or daily practice	Light experience excerpts
99000	M	Vipassan 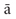 (Bur), Shamatha (Bur)	7	Retreat	I started getting these meditative states that were like seeing a curtain of light, so even in a dark room meditating at night there would be a sense of – as if there were lights on in the room, and when I opened my eyes there wouldn’t be, but…there was often a curtain, this internal curtain of light. I began to have very luminous imaginations where…if I pictured something it was as if I was actually there, so when I pulled back a memory or a fantasy of being on a beach my mind became so bright it was as if I was actually there, I could see all the details of all the trees and hear the birds calling and… it was as if I was actually standing at the beach. So this very luminous mind… was coming a lot also when I was sleeping; I was getting incredibly vivid dreams… I would be walking around my cabin, and after ten-fifteen minutes of this I realized I was still dreaming, but it was so incredibly vivid as a first-hand experience. This is all talked about in the developing of concentration.
99003	M	Vipassan 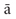 (USA)	5	Retreat	Even with my eyes closed, there would be a lot of light in the visual frame, so to speak. Diffuse, but bright… My eyes were closed – there was what appeared to be a moon-shaped object in my consciousness directly above me, about the same size as the moon if you lay down on the ground and look into the night sky. It was white. When I let go I was totally enveloped inside this light… I was seeing colors and lights and all kinds of things going on... Blue, purple, red. They were globes; they were kind of like Christmas tree lights hanging out in space except they were round. They were very distinct; they weren’t fuzzy, they were very clear.
99005	M	Zen	8	Daily	So most people see that if they do eyes open meditation (they have) the sense that reality is pixelating a little bit and becoming very very vivid… My personal experience of it... definitely the Christmas lights and definitely the shimmering… my tendency is to perceive it that way. The Christmas lights (…were) almost like small radiant bursts.
99008	M	Vipassan 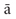 (Bur)	2	Retreat	The mind became very strong and bright. By the end of the retreat, it was just on, the lights were on all the time... The last night, I sat the whole night (with) no effort just all kinds of stuff would be happening, energetic stuff, painful things, it’s just the mind was totally unmoving.
99009	F	Vipassan 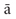 (USA)	2	Retreat	I was just bursting with light, I would just close my eyes and it was just brilliant light. I just felt like I was radiating, like there were rays of light coming out of me. … It felt like it was just emanating from my body and my system. But this wasn’t my entire retreat by any means, it was just near the end.
99011	M	Vipassan 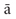 (USA)	6	Daily	And…I had the lights out, so I had this sensation of lights passing over my eyes, but they weren’t external. … It seemed to be something just happening to my inner eye, or maybe on my retina, or something when you close your eyes and become deeply relaxed and you start seeing these sort of pleasant pulsations of color, of various forms of color. I think it was that, but like times one hundred, it was just all sped up to a ridiculous degree… It created this perceptual distortion which I felt like I was in a tunnel, or in some kind of train, and there were lights passing me as I moved forward. It was almost as if I was being carried forward…lying on my back through some sort of…crazy roller coaster ride.
99019	F	Shamatha (Tib)	5	Retreat	Sometimes there were, oftentimes just a white spot, sometimes multiple white spots, sometimes the spots, or “little stars” as I called them, would float together in a wave, like a group of birds migrating, but I would just let those things come and go. All of a sudden this tremendous amount of bliss, the jet engine feelings of bliss, would come up while I was sitting and meditating. Or the white lights, or sometimes blue lights, would come up while I was meditating. It was not associated with an unpleasant feeling, or with any kind of palpitations, or rushes, other than this energetic jet engine vibration feeling.
99021	M	Vipassan 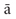 (USA)	5	Daily	I’ve put all of these experiences more in the arising and passing part of the path… golden light that fills the sky and my body feeling like the same nature as that, and the combining and unitive kind of experience, but also there was one night…where my body just was breaking apart into sparkles and like electrical sparks being sent off everywhere in all directions with…so that kind of light and physical…so that the sparks one is obviously more dynamic, the gold one is more unitive, and then during meditation there’s plenty of states where you’ll see jewel lights or blues and greens and oranges and that seems to be associated with being fairly concentrated at that point.
99022	M	Zen	5	Daily	I’ve seen ropes of shimmering… where it’s causing space behind it to shimmer a little bit and move. I’ve occasionally had the visual field get much brighter than normal for no particular reason (and) I’d say that’s attached to concentration of some sort… In concentration I’ve had rays of white light that go through everything. They’re either coming from behind me somewhere or coming out of the object that I was concentrating on. … I saw it with my eyes open and it wasn’t really seeing it was something else, even though I still was perceiving that it was there.

## DISCUSSION

Our discussion begins by presenting a basic typology of meditation-induced light experiences based upon the data derived from the coded interviews from our subject pool. Data from these first-person reports of meditation-induced light experiences are then compared to typologies of related phenomena derived from traditional Buddhist literary sources. We also present traditional Buddhist interpretations of their significance in terms of the practitioner’s progress in meditation.

The second main subsection discusses the neurobiology of light-related experiences according to scientific research on sensory deprivation, perceptual isolation, and clinical disorders of the visual system. We draw upon this literature both to posit the possible underlying mechanisms of meditation-induced light experiences and in order to suggest a novel interpretation of meditation that calls attention to its structural similarities with sensory deprivation and perceptual isolation.

We end our discussion with the implications of our findings for the scientific study and clinical application of meditation.

### A TYPOLOGY OF MEDITATION-INDUCED LIGHT EXPERIENCES BASED UPON QUALITATIVE DATA AND TEXTUAL SOURCES

#### Class one: discrete lightforms

Four practitioners reported discrete lightforms appearing as either “globes,” “jewels,” or “spots.” These lights appeared in various colors and were also described as being “very vivid” or “very distinct.” Discrete lightforms could be either singular or multiple in number and were generally small, being described as “little stars” or “small radiant bursts.” Some of these bright, luminous shapes were characterized by some practitioners as being stable, or as “hanging out in space,” whereas for others they were more animated. For example, one practitioner described spots of lights as floating “together in a wave, sort of like a group of birds migrating.” Small lights – singular or multiple – that appear as generally round points of colored light are the first group of phenomena that can be linked with accounts derived from traditional Buddhist literature.

Literature throughout the Therav
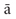
da Buddhist tradition describes a particular mental phenomenon called a *nimitta*. The *nimitta* arises once a preliminary mastery of concentration has been established, and especially through developing concentration by focusing attention on the inhalation and exhalation of the breath (Ledi Sayadaw, 1999). Although the particular mental image or form of the *nimitta* varies across practitioners and depends upon the object of concentration, the most common Buddhist meditation practice involves taking the breath as a primary object of attention. A fifth century treatise entitled *The Path of Purification* (Buddhaghosa, 1999, p. 277) describes the *nimitta* that arises through concentration on the breath as follows:

It appears to some as a star or cluster of gems or a cluster of pearls, […] to others like a long braid string or a wreath of flowers or a puff of smoke, to others like a stretched-out cobweb or a film of cloud or a lotus flower or a chariot wheel or the moon’s disk or the sun’s disk.

Many of these initial descriptions of the *nimitta* are consonant with the reports of small points of light described as “globes,” “jewels,” or “stars” discussed above. One practitioner from our study also described a white light that appeared to be the size of moon as seen in the night sky.

While Buddhaghosa suggests that the different lightforms of the *nimitta* are idiosyncratic, Buddhist sources from other traditions present similar phenomena to the “cluster of gems,” “puff of smoke,” and “film of cloud” as arising in a progressive sequence*.* Extensive presentations of light-related signs of attainment can be found in the Buddhist *tantras* – a vast body of literature associated with ritual and contemplative practices that developed and flourished in India between the seventh and the eleventh century (Samuel, 2008). According to a traditional commentary on the *tantras*, a typical trajectory for the lightforms is as follows: The first is described as being “like seeing a mirage,” the second as a “smoke-like vision,” the third a “vision like flickering fireflies,” the fourth is like “the glow of a butterlamp,” and the fifth is “like a clear autumn sky pervaded by the light of the full moon” (Dondrup, 1997, p. 85). Various *tantras* present the progressive sequence of mental images in different orders; Gyatso (2004) explains that this is due to the different techniques that can be used to induce the involuntary mental images.

While the reference in the *tantras* to lights “like flickering fireflies” closely resembles the reports of “Christmas lights” and “small radiant bursts” discussed in the first class of light-related phenomena, the depictions of a mirage-like light and an illuminated autumn sky more closely correspond to phenomena we include in the second class of meditation-induced light experiences.

#### Class two: patterned and diffuse lights

The second class of light-related phenomena includes patterned and diffuse changes to the visual field, most commonly described as shimmering, pixelation, or brightening. This class of phenomena can be distinguished from the first class on account of not having a distinct and circumscribed shape, size, color, or spatial location. Instead, they are characterized as being superimposed on the practitioner’s perception of space, and often arise in conjunction with the perception of external objects.

***Shimmering.*** Like the tantric visions of seeing a mirage, one practitioner described space as “shimmering,” and alternately as seeing “ropes” that would emerge from objects in space and cause the space behind it “to shimmer a little bit and move.” Contemporary authors in a Tibetan Buddhist lineage explain that “when you meditate, many different lights can appear [including…] all kinds of shimmering experiences,” and these are taken to be “good signs” (Sherab and Dongyal, 2012, p. 94).

***Pixelation.*** Another example of an alteration of the visual field comes from a practitioner who described the visual field as “pixelating a little bit and becoming very very vivid.” A similar cluster of phenomena that co-arise with the perception of external objects include reports of “seeing energy instead of seeing solid objects” and “seeing rays of light that go through everything.” In a description strikingly parallel to “pixelating,” Tibetan author Dudjom Lingpa includes “the perception of all phenomena as brilliantly colored particles” among the list of meditation experiences (*nyams*) that can arise from proficiency in concentration practice (Wallace, 2011, p. 136). Full et al. (2013) documented advanced meditators in a Burmese Therav
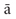
da tradition, some of whom also reported perceiving both external objects and their own body as small particles as a result of directing a concentrated mind toward the investigation of the body.

***Brightening.*** In addition to the shimmering and pixelation described above, six practitioners reported a homogenous brightening of the visual field. Two of these practitioners reported that the visual field was brighter when the eyes were open, including a report of a “golden light that fills the sky,” which is quite similar to the imagery used in the Buddhist *tantras* to characterize the fifth sign of attainment.

Four other practitioners characterized the homogenous field of light as a mental image arising behind closed eyes. For example, one practitioner described an “internal curtain of light” that would be most apparent when meditating in a dark room or with eyes closed. On account of this curtain of light, this practitioner reported being able to perceive memories and dream-like reveries as clearly as external objects. This increasing brightness of the visual field may again be related to the fifth, unstructured “sky-like” sign of attainment presented in the *tantras*, or, it may be closer to a phenomena described in the canonical sources of early Buddhism as the “pure bright mind.” This “pure bright mind” is associated with the fourth state of meditative absorption (*jh*
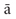
*na*) attained through the cultivation of concentration. In one passage, this quality of awareness pervades the body in a manner compared to a man “sitting covered from the head down with a white cloth, so that there would be no part of his whole body unpervaded by the white cloth; so too, a [monk] sits pervading this body with a pure bright mind” (Nanamoli and Bodhi, 1995, p. 369).

Two practitioners also reported a proprioceptive dimension to their meditation-induced light experience. One practitioner explained that “my body just was breaking apart into sparkles and like electrical sparks being sent off everywhere in all directions”; the other “felt like I was radiating, like there were rays of light coming out of me.” In contemporary Therav
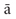
da accounts (Sayadaw, 2010, p. 122), concentration directed toward the body is similarly associated with seeing “a smoky gray light [that will] become whiter like cotton wool, and then bright white, like clouds,” and furthermore with seeing the body “sparkle and emit light.” Experiences of light emanating from the meditator’s body are also associated with a calm mind and with a particular stage of insight called the “knowledge of arising and passing away” (Nanarama, 1983, p. 36), the significance which is discussed below.

#### Textual interpretations of meditation-induced light experiences

As suggested above, reports of meditation-induced light phenomena can be found across Buddhist traditions, in both historical, textual accounts and among accounts from contemporary practitioners. A survey of both historical and contemporary accounts reveals that there is no single, consistent interpretation of meditation-induced light experiences in Buddhist traditions. Some types of light may signal that a particular discipline such as concentration has reached a certain stage of development, whereas other lights may be the result of imbalanced practice. Some interpret lights as a vehicle for investigating the constructed nature of phenomenal appearances; other light experiences are deemed unimportant side effects of meditation.

In the context of Therav
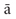
da and Tantric Buddhism, meditation-induced light experiences are often deliberately sought as part of the method of transforming consciousness or are interpreted as a sign that such transformations have occurred. According to typical Therav
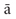
da instructions on concentration practice, such as those found Buddhaghosa’s *The Path of Purification*, once a *nimitta* arises, it is take as a sign that an initial stage of meditative absorption has been reached. At that point, this involuntary light experience can then replace the breath or an external object as the new object of concentration (Ledi Sayadaw, 1999). Similarly, in Tibetan traditions, the progressive sequence of signs that are thought to manifest on account of tantric practices, including both discrete lightforms and diffuse lights such as shimmering, are interpreted as positive signs that the clarity and luminosity of the mind is becoming apparent to the practitioner (Gyatso, 2004; Sherab and Dongyal, 2012).

However, similar typologies of light-related experiences are elsewhere treated as involuntary side effects of meditation. Some Therav
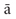
da Buddhist authors from contemporary Burmese lineages (Nanarama, 1983, p. 36; Sayadaw, 1994, p. 13–14) identify the arising of a “brilliant light,” a “flash of lightning,” a “lamplight in the distance,” or a “light [that] emanates from [the meditator’s] own body” as “illumination” experiences – one of 10 “imperfections of insight” that can arise during a stage of practice called the “knowledge of arising and passing away.” These light-related meditation experiences, while still taken as signs of a calm mind capable of carefully investigating present moment experience, are nevertheless treated as “corruptions” or “imperfections” because they are so enticing that they can lead the meditator astray from the practice instructions. Similarly, the phenomena classified in Tibetan Buddhism as “meditation experiences” (*nyams*) also include light-related experiences among the range of phenomena that can arise in meditation. Like the lights that arise in the context of the imperfections of insight, light-related *nyams* such as pixelation are to be left alone, and the practitioner is to proceed without becoming attached to them (Wallace, 2011). In Zen Buddhist traditions, the term *makyo* is used to refer to a similar category of “side-effects” or “disturbing conditions” that can arise during the course of practice (Austin, 1999, p. 373). Consonant with Southeast Asian Therav
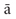
da and Tibetan Tantric Buddhist sources, Sogen (2001, p. 84) associates the arising of *makyo* with “proof of considerable maturity in Zen concentration.” However, “even if a glorious light shines, […] they all belong to *makyo* regardless of whether they are good or bad,” and the practitioner should respond by “paying no attention to them” (p. 87).

According to other traditional interpretations, especially prominent in the Dzogchen tradition of Tibetan Buddhism, one of the objectives of advanced meditation practices is to stabilize the lightforms that arise and investigate them (Chagme and Gyatrul, 2000, p. 159; Namdak, 2006, p. 197). Temple wall paintings featuring both descriptions and representations of these lightforms describe a trajectory beginning with “countless minor circles like pearls on a string” that develop into visions of “luminous spheres, grids, and disk of light” (Baker, 2000, p. 119). Such meditation-induced light experiences are not important in and of themselves; rather, they are valuable only insofar as they assist the practitioner in recognizing the way in which the mind constructs visual appearances and reifies them as external objects (Wangyal, 2002, pp. 131–133; Namdak, 2006, p. 198). In this trajectory of practice, certain Buddhist meditation traditions utilize visual hallucinations as a means of gaining insight into the way in which perceptual experience of the phenomenal world is constructed, rather than given. Some contemporary theories in cognitive science have characterized phenomenal consciousness as a process that simulates the relationship between the body and its environment (e.g., Metzinger, 2003; Revonsuo, 2006) in a manner compatible with certain Buddhist approaches to insight (Waldron, 2006). As the following section will demonstrate, the neurobiology of visual hallucinations and veridical visual perceptions are closely related (Ffytche et al., 1998; Lloyd et al., 2012).

### NEUROBIOLOGICAL PERSPECTIVES ON LIGHT-RELATED EXPERIENCES

Scientific studies of light-related experiences tend to classify such phenomena as visual hallucinations. This section presents findings from sensory deprivation, perceptual isolation, and disorders of the visual system.

#### Sensory deprivation

Sensory deprivation includes exposure to environments that present the subject with minimal sensory input. Through darkness, silence, isolation, and bodily stillness, respectively, the subject’s visual, auditory, social, and kinesthetic experience is reduced as much as possible (Zubek et al., 1961; Rossi, 1969; Merabet et al., 2004; Kjellgren et al., 2008; Mason and Brady, 2009). Sensory deprivation may include occlusion of individual sense organs, such as with earplugs or blindfolds, or multiple senses at once, such as through sitting alone in a dark and silent room.

#### Perceptual isolation

Perceptual isolation refers to exposure to homogenous, invariant, or unstructured stimuli (Wackermann et al., 2002,^2008^; Pütz et al., 2006; Lloyd et al., 2012). While sensory input is not technically absent in perceptual isolation, the monotony leads to habituation where input is “filtered out,” which can mimic the effects of decreased input of sensory deprivation (Pütz et al., 2006).

#### Visual impairment

Among disorders of the visual system, Charles Bonnet Syndrome is classically associated with visual hallucinations and light-related experiences. Charles Bonnet Syndrome is most commonly found among elderly patients who have very poor vision or are blind on account of an impairment of their visual system, ranging from eye abnormalities to dysfunctions in the occipital lobe (Vukicevic and Fitzmaurice, 2008; Kazui et al., 2009).

All three of these conditions – sensory deprivation, perceptual isolation, and Charles Bonnet Syndrome – are characterized by impaired sensory input to the visual system, and all result in the rise of involuntary visual hallucinations, where “hallucinations” are defined as a “percepts, experienced by a waking individual, in the absence of appropriate stimuli from the extracorporeal world” (Blom, 2013, p. 44). Hallucinations are different both from illusions, which are distortions of actual external stimuli, and from intentionally constructed mental imagery, which remains under volitional control and lacks perceptual vividness (Reichert et al., 2013).

While the sensory loss that occurs at sleep onset may also result in hallucinations, these “hypnagogic” hallucinations are more similar – both neurologically and phenomenologically – to dreams that occur during REM sleep than to visual hallucinations that occur during wake (Wackermann et al., 2002; Collerton and Perry, 2011; Fenelon, 2013). Like dreams, hypnagogic hallucinations tend to occur in multiple sensory modalities at once, are panoramic or “full screen” rather than circumscribed, and are associated with lack of insight and strong, often negative affect (Cheyne et al., 1999a,b; Ohayon, 2000; Collerton and Perry, 2011). Thus, our typologies of visual hallucinations will focus on those that arise during wake, as the phenomenology of waking visual hallucinations is the most congruent with the reports from participants in our study.

### SCIENTIFIC TYPOLOGY OF VISUAL HALLUCINATIONS

Researchers of sensory deprivation (Zubek et al., 1961; Zuckerman, 1969; Merabet et al., 2004), perceptual isolation (Wackermann et al., 2002,^2008^; Pütz et al., 2006; Lloyd et al., 2012), and visual disorders (Santhouse et al., 2000; Wilkinson, 2004; Ffytche et al., 2010) have developed similar typologies of visual hallucinations. Documented visual hallucinations range from simple forms or flashes of light or color to grid-like patterns to animated figures or scenes. While “complex” hallucinations include faces, objects, and landscapes, light-related experiences of both discrete lightforms and patterned or diffuse lights fall into the category of “simple hallucinations.” As explained above, our present analysis is limited to “simple” visual hallucinations.

### SIMPLE HALLUCINATIONS

Simple hallucinations often include circumscribed objects, patterns, and diffuse changes across the visual field. Circumscribed hallucinations include points of light, colored lights, or shapes (phosphenes), or flashes of light (photopsia) sometimes described as “dots” or “stars.”

Patterned hallucinations may include regular, overlapping patterns (tessellopsia) like lattices, grids, and cobwebs, branching forms (dendropsia) like vines, ropes or roads, and zigzag patterns (teichopsia). Other patterns include the perception of visual snow or television-like static. Diffuse hallucinations include a brightening of the visual field, descriptions of mist or fog, shimmering, or bright sunsets (Ffytche and Howard, 1999; Merabet et al., 2004; Wilkinson, 2004; Ffytche et al., 2010; Lloyd et al., 2012; **Table 2**).

**Table 2:** Comparative typology of visual hallucinations and meditation-related light experiences.

Typologies derived from sensory deprivation, perceptual isolation, and disorders of the visual system	Typologies derived from qualitative reports from contemporary practitioners of Buddhist meditation	Typologies derived from Buddhist literary sources
**Class 1: Discrete lightforms**		
Phosphenes: colored spots, dots, or points of light	Blue, purple, and red globes	Cluster of pearls
	Jewel lights	Cluster of gems
	Christmas tree lights	Luminous spheres
	White or blue lights	Lamplight in the distance
	White spots or little stars	A star
Photopsia: flashes of lights	Small radiant bursts	Flickering fireflies
Sun’s disk and sunset	Moon-shaped object	Moon’s disk
		Sun’s disk
**Class 2: Patterned and diffuse lights**		
Teichopsia: zigzag colors		Stretched-out cobweb
Tessellopsia: lattice, nets, grids, or cobwebs		Grids
Shimmering	Ropes of shimmering	Shimmering experiences
Mist	Seeing energy instead of solid objects	Mirage
Luminous fog		Film of cloud
		Smoke-like vision
Visual snow, television-like static	Reality is pixelating	All phenomena as brilliantly colored particles
	Electrical sparks in all directions	Perceiving body and objects as small particles
	Rays of light that go through everything	
Lighter visual field	Brighter visual field	Glow of a butter lamp
	Lights always on	
	Curtain of light	Clear autumn sky pervaded by the light of the full moon
	Golden light that fills the sky	
Bright sunsets	Body feeling like the same nature as light	Pervading the body with a pure bright mind
	Bursting with light	
	Light emanating from the body	Light emanates from his own body
	Bright mind	

Both circumscribed and diffuse simple visual hallucinations arise as result of sensory deprivation (Zubek et al., 1961; Zuckerman, 1969; Merabet et al., 2004) and perceptual isolation (Wackermann et al., 2008; Lloyd et al., 2012). In some cases of perceptual isolation, the visual field may disappear entirely, leaving subjects “uncertain whether their eyes were open or closed, or even unable to control their eye movements. In the ‘luminous fog’ of the (homogenous visual field) the subjects do not see anything; in the ‘blank-out’ periods, they may experience the presence of ‘nothingness’;” (Wackermann et al., 2008, p. 1367). The authors also point out that natural environments, such as a uniformly blue or cloudy sky, can function in a manner similar to intentionally constructed perceptual isolation environments (p. 1365), and that depending upon the subject’s disposition, perceptual isolation experiences and especially “blank out” episodes “may elicit even mystical or religious interpretations” (p. 1368).

Simple hallucinations are much more common than complex ones. Among those with disorders of the visual system, more than 50% of visually impaired individuals report simple hallucinations, while only 15–25% report complex hallucinations (Menon et al., 2003; Vukicevic and Fitzmaurice, 2008). Simple hallucinations can be evoked with very brief exposure to attenuated input, whereas complex hallucinations require more prolonged or more extensive sensory deprivation or perceptual isolation (Wackermann et al., 2002,^2008^; Pütz et al., 2006; Lloyd et al., 2012). Complex hallucinations are thought to draw upon many brain areas, including those involved in memory (Collerton et al., 2005), and require more widespread and extensive neuroplastic modifications (Ffytche et al., 2010). More prolonged or extensive deprivation and brain changes may also begin to include cross-modal experiences where visual phenomena are experienced proprioceptively and incorporated into body schema and emotional meaning or value structures (Ffytche et al., 2010).

Researchers have found that simple visual hallucinations are associated with activity in the occipital cortex, the primary visual-processing center of the brain (Boroojerdi et al., 2000; Merabet et al., 2004). Ffytche et al., (1998, p. 740) presents fMRI-based evidence of a specific “correlation between the location of activity within a specialized cortex and the contents of a hallucination.” Visual hallucinations of grid-like patterns, for instance, correlated with activity in the collateral sulcus, and color hallucinations correlated with activity in the fusiform gyrus. This led the researchers to conclude that in terms of their neurobiology, visual hallucinations are more like ordinary perceptions than they are like visualized mental images. Similarly, a perceptual isolation study (Lloyd et al., 2012) demonstrated that subjects responded to and could modulate their attention in relation to visual and auditory hallucinations as they would with ordinary perceptions.

### WHY SENSORY LOSS CAUSES HALLUCINATIONS

Attenuation of sensory input reliably leads to hallucinations, even after a short time. Decreased sensory input leads to spontaneous firing and hallucinations through homeostatic plasticity – a set of feedback mechanisms that neuronal circuits use to maintain stable activity and firing rates close to a set point (Desai, 2003). Homeostatic plasticity may include adjusting synaptic input strength (synaptic homeostasis) or changing the intrinsic excitability of the neuron (intrinsic plasticity) (Turrigiano, 2011). When sensory inputs are attenuated or lost, the homeostatic plasticity mechanisms increase neuronal excitability and firing thresholds (Boroojerdi et al., 2000; Fierro et al., 2005; Pitskel et al., 2007), which may be experienced as heightened sensory acuity or perceptual sensitivity (Suedfeld, 1975). In the case of sensory attenuation, homeostatic mechanisms often overcompensate to the point of generating spontaneous firing, which is experienced subjectively as hallucinations (Schultz and Melzack, 1991; Burke, 2002; Maffei and Turrigiano, 2008; Reichert et al., 2013).

A wide variety of conditions of sensory attenuation or monotony lead to increased cortical excitability, spontaneous firing, and hallucinations. Given that meditators are reporting visual hallucinations in the context of meditation, it is worth considering the sensory attenuating qualities of meditation practices.

### BUDDHIST MEDITATION AS A FORM OF SENSORY DEPRIVATION AND PERCEPTUAL ISOLATION

#### Structural components

While there are a variety of approaches to meditation, many practices incorporate structural components analogous to sensory deprivation and perceptual isolation, including sensory, social and kinesthetic deprivation, or invariance. The practice of meditation tends to be done in social isolation or in groups in which social interactions are minimized. During a formal practice session, practitioners adopt a stable, seated posture. The locations for meditation practice also tend to be quiet environments removed from excessive auditory stimuli. Through dimly lit environments or through practicing with the eyes closed or open with a fixed gaze, visual stimuli are restricted.

It is important to note that even when meditation is not practiced within such sensory minimal environments, the *practice* of meditation functions in a manner analogous to perceptual isolation through restricting attention to monotonous or homogenous stimuli. In practices that involve movement, the emphasis is on monotonous, repetitive movements, such as slow walking. In concentration practice on the breath, other kinesthetic, auditory, and visual stimuli are deselected in order to attend, again and again, to the repetition of inhalation and exhalation. Even when meditators practice with their eyes open, the gaze either remains unfocused and on the entirety of the visual field or is restricted to a single invariant object.

Thus, whether through practicing in environments with minimal sensory input, or through attending only to monotonous, repetitive stimuli, the context and function of meditation is similar to both sensory deprivation and perceptual isolation (**Table 3**). That advanced practitioners often deliberately choose to isolate themselves further by practicing in remote caves or in sensory deprivation environments demonstrates how Buddhist meditation traditions have recognized the importance of practicing within sensory minimal environments. For example, the Tibetan Buddhist practice of “dark retreat” (*mun mtshams*) suggests that deliberate and intensive sensory deprivation is thought to be particularly effective in producing profound shifts in perception and cognition (Chagme and Gyatrul, 1998; Wangyal, 2000,^2002^; Gyatso, 2004; Reynolds, 2011). Not surprisingly, discussions of meditation experiences in dark retreat closely resemble the typology of visual hallucinations discussed above. Tenzin Wangyal Rinpoche, who undertook an extensive dark retreat when he was a young boy, reports both simple and complex visual hallucinations arising as a result of the prolonged sensory deprivation (Wangyal, 2000,^2002^, pp. 32, 132). Tibetan Buddhists also prescribe a shorter-term technique of fixing the gaze upon a cloudless sky as a means of inducing simple visual hallucinations (Gyatso, 2004). As mentioned above, Wackermann et al. (2008) suggest that a uniform sky serves as a natural perceptual isolation condition.

**Table 3:** Comparison of sensory deprivation, perceptual isolation, and meditation.

	Sensory deprivation	Perceptual isolation	Meditation
**Social**	Isolation from human interaction and communication	Isolation from human interaction and communication	Retreat environment temporary social isolation
**Kinesthetic**	Immobilization, floatation tank	Restricted to comfortable chair	Stable, seated posture, monotonous movements
**Auditory**	Silence, soundproof room, earmuffs & Headphones with white noise monotonous sound, consistent volume	Quiet retreat environment, redirection of attention away from sound
**Visual**	Darkness, blindfolds	Eye shields and lighting create homogenous visual field	Eyes closed, gaze fixed on single object, gaze directed at space

#### Concentration as sensory deprivation

In addition to the structural aspects of meditation practices listed above, it is possible that the intense attentional engagement of Buddhist meditation practices also functions as a form of sensory deprivation. By “guarding the sense doors,” some meditation practices aim to limit the sensory input impinging on awareness through restricting attention to a single object of perception, such as a visual object or the breath (Buddhaghosa, 1999; Dalai Lama, 2001). Whether in “focused attention” types of meditation, where one object is continually selected, or in “open monitoring” meditation, where many different objects are selected sequentially (Lutz et al., 2008), attention facilitates the processing of relevant information by suppressing irrelevant sensory inputs (Briggs et al., 2013), and can therefore be viewed as a largely inhibitory process (Kerlin et al., 2010; Foxe and Snyder, 2011).

The act of paying attention facilitates the processing of relevant stimuli while inhibiting irrelevant stimuli. In terms of the brain’s electrical activity, fast excitatory frequencies and slow inhibitory frequencies interact to facilitate attention (Jensen and Mazaheri, 2010). The active processing of relevant stimuli is associated with increased fast frequency (gamma) oscillations and decreased slow frequencies (i.e., decreased alpha power or increased alpha desynchronization) in the engaged areas (Pfurtscheller and Lopes da Silva, 1999). In contrast, suppression or inhibition of irrelevant stimuli is associated with the opposite pattern, decreased beta and gamma and increased alpha power or alpha synchronization in areas not related to the task (Pfurtscheller and Lopes da Silva, 1999; Suffczynski et al., 2001; Lutz et al., 2007; Kerr et al., 2011). Alpha activity is also associated with decreases in fMRI BOLD signal and is thought to reflect the functional inhibition of neural activity in task-irrelevant areas (Goldman et al., 2002; Feige et al., 2005). For example, during visual tasks, information from non-visual (e.g., motor) areas is inhibited via increased alpha power (Pfurtscheller, 1992). Studies of spatial attention have shown decreases in alpha power in areas related to active processing of target locations but increases in alpha in areas related to non-target locations (Thut et al., 2006; Rihs et al., 2007; Kerr et al., 2011). Although most early research on attention focused on gamma activity in task-relevant areas and ignored alpha activity in task-irrelevant areas, it now appears that alpha inhibition is as important or more important than gamma facilitation. Indeed, optimal task performance on a number of cognitive tasks, including selective and sustained attention, is determined by the extent of alpha activity in task-irrelevant areas rather than gamma in task-relevant areas (Dockree et al., 2007; Jensen and Mazaheri, 2010). Similarly, widespread increases in alpha power in meditators (Cahn and Polich, 2006) that were initially viewed as “idling” (Pfurtscheller et al., 1996) or relaxation (Fenwick et al., 1977) are now thought to reflect the active inhibition of irrelevant cortical inputs as a means of facilitating attention (Jensen and Mazaheri, 2010; Britton et al., 2013; Kerr et al., 2013).

Various meditation practices, especially focused attention or concentration practice, involve selecting target or relevant stimuli and deselecting non-target or irrelevant stimuli. Areas of the body that are related to the sensations of breathing or walking are a common target of focus in many forms of Buddhist meditation, and the ability to maintain focus on these target areas and inhibit distracting non-target stimuli is considered to be a hallmark of proficiency in focused attention forms of meditative practice. Meditation-related increases in interoceptive accuracy to frequently attended targets (Kerr et al., 2011; Silverstein et al., 2011; Fox et al., 2012) are associated with longer lifetime meditative experience (Fox et al., 2012) and are thought to be determined by the extent of alpha-modulated inhibition in non-target areas (Kelly et al., 2009; Jones et al., 2010; Foxe and Snyder, 2011). Thus, it could be argued that proficiency or expertise in focused attention as a result of meditation can be indexed by the degree of cortical inhibition.

Increased throughput of frequently attended-to “target” areas (e.g., improved interoception) is a straightforward example of experience-dependent neuroplasticity that underlies successful skill acquisition. However, the model in this paper suggests that inhibition of non-target sensory input can also result in a compensatory increase in neuronal excitability, which is often measured either by a decreased sensory threshold or by increased firing rates or spontaneous firings (hallucinations). In terms of decreased sensory thresholds, studies of Tibetan Shamatha (MacLean et al., 2010) and Therav
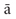
da Vipassan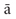 (Brown et al., 1984) practitioners who were not using visual objects as their primary target have found long-lasting (more than 5 months) decreases in visual perception thresholds. In terms of increased firing rates, long-term practitioners in both Therav
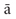
da Vipassan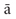 (Cahn et al., 2010; Ferrarelli et al., 2013) and Tibetan Shamatha (Ferrarelli et al., 2013) traditions were found to have unexplained increases in occipital gamma during meditation and NREM sleep that was associated with lifetime expertise in meditation. The reports of visual hallucinations from this paper suggest that visual areas of the occipital cortex have become hyperexcitable as a result of focused attention on non-visual target areas.

While it is still unknown if meditation-related light experiences are indeed caused by suppression of sensory input via alpha inhibition leading to compensatory disinhibition, this model is supported by perceptual isolation and sensory deprivation studies. Visual hallucinations in perceptual isolation (Wackermann et al., 2002; Pütz et al., 2006) and in sensory deprivation (Hayashi et al., 1992) are preceded by increases in global alpha power, followed by sudden high frequency EEG at occipital sites just before the hallucination (Pütz et al., 2006). Although the relationship to a *visual* hallucination is unclear, Lo et al. (2003) found a similar progression in EEG power among meditators who reported an experience of “inner energy” or “inner light.”

In support of concentration playing a role, it is worth noting that seven of the nine practitioners who reported lights (78%) spontaneously connected their meditation-induced light experiences with a period of increased concentration, a claim also made in Buddhist literature across traditions. This association fits with existing neurobiological models that suggest that hallucinations can be related to alpha inhibition (Hayashi et al., 1992) and to the “attentional spotlight” of concentrated attention (Aleman and Laroi, 2008, p. 173). Together these data suggest that the attentional and structural components of meditation serve to attenuate sensory input, which activates homeostatic forms of neuroplasticity that lead to hyperexcitability, spontaneous firing, and hallucinations.

### IMPLICATIONS

#### Implications for clinical neuroscience

The possibility of viewing meditation practice as a form of sensory deprivation has potentially profound implications. Current medical technologies are combining non-invasive brain stimulation techniques that alter neuronal excitability and enhance cortical plasticity with training protocols to enhance outcomes in neuropsychiatric patients, including dementia, pain, addiction, anxiety, and depression (Nitsche et al., 2008; Halko et al., 2011; Kuo et al., 2013). In addition to improving symptoms, this enhanced neuroplasticity is also associated with improved learning, working memory, attention, and other cognitive improvements (Guse et al., 2010). Similarly, the attenuation of sensory inputs increases neuronal excitability and facilitates a period of enhanced neuroplasticity (Ffytche et al., 1998; Boroojerdi et al., 2000,^2001^). Whether through brain stimulation or sensory attenuation, changes in neuronal excitability that accelerate neuroplasticity can be used to facilitate therapeutic changes beyond usual training protocols. Since meditation training contains sensory attenuation components, it is possible that this form of cognitive training may have enhanced neuroplastic potential. Furthermore, the appearance of visual lights or other hallucinations could potentially serve as an indicator of a period of enhanced neuroplasticity, during which the ability to make a significant affective, perceptual, or cognitive shifts could be maximized.

#### Implications for the scientific study of meditation

Current researchers assert that “the mental training of meditation is fundamentally no different than other forms of skill acquisition that can induce plastic changes in the brain” (Davidson and Lutz, 2008, p. 176). However, the meditation-induced light experiences described in this paper suggest that meditation is a form of sensory attenuation that is capable of activating an enhanced period of neuroplasticity that may not occur in other forms of skill acquisition. Visual hallucinations arising in the context of meditation practice may serve as indicators that homeostatic plasticity has been activated and that the brain may be more malleable to learning and change. While still highly speculative, this suggests that the sensory attenuation components of meditation may enhance its neuroplastic potential beyond other forms of skill acquisition.

Meditation researchers are also currently struggling with ways to measure meditative proficiency or expertise. Current attempts to measure expertise include self-reported “mindfulness” scales and the estimated number of hours of practice, both of which are problematic (Grossman and Van Dam, 2011; Van Dam et al., 2012). Meditation-induced light experiences are worthy of further consideration and study as a potential indicator of meditative proficiency. Converging reports from our subjects, Buddhist textual sources, as well as multiple scientific research domains suggest that lights may, at least in some cases, be signs that the practitioner has attained a certain degree of concentration. Proficiency in concentration could be determined by the degree to which a practitioner is able to inhibit irrelevant inputs from impinging on attention. Meditation-related light experiences may serve as a fairly consistent signpost of concentrative attainment across Buddhist traditions because such visual hallucinations tend to arise as a result of the attenuation of sensory input. In addition, because the spontaneous firings that generate visual hallucinations are associated with the activation of homeostatic plasticity, they may also herald entry into a time of enhanced learning, progress, and insight. However, any traditional or well-known marker of progress potentially introduces demand characteristics. Further research that triangulates self-reports with both behavioral and neurobiological markers is necessary.

#### Implications for clinical applications of meditation

While light-related experiences arising in the context of meditation are well documented in traditional contexts, they are largely unknown in clinical settings. In assessing meditators practicing outside of traditional contexts, it is important to carefully attend to the nuances of light-related discourses when evaluating whether lights are signs of positive changes or inconsequential side effects of meditation. In traditional contexts, meditation-induced light experiences are frequently subject to scrutiny before they are attributed either positive or negative value. Without the traditional safeguards of a student-teacher relationship, the clinical application of meditation practices may be particularly susceptible to misinterpreting lights and other meditation experiences, since visual hallucinations are well-known indicators of both psychosis and vision system impairment. It is important, therefore, not to uncritically pathologize these anomalous perceptual experiences. Light-related experiences are likely to be benign, but may cause distress to the practitioner if they are unexpected or accompanied by other psychological changes. By empirically studying and documenting meditation-induced light experiences and describing them within their traditional Buddhist frameworks, we hope to help educate clinicians and meditation teachers about some of the common side effects of meditation in order to create more appropriate support structures for practitioners.

#### Study limitations and suggestions for future research

The investigation of phenomena that has received little empirical attention requires inductive methods that are intended to be free of both assumptions and hypotheses. Thus, our grounded-theory-based approach, which is appropriate for this stage of research, is both a strength and a limitation. As a strength, the open-ended approach, which discourages researchers from asking specific questions that would bias the subjects’ answers, helps to minimize demand characteristics of the interview. As a limitation, by not asking participants if they had certain experiences, such as lights, the current report may have underestimated the actual prevalence of light-related meditation experiences. Our current approach can only answer the question “What types of experiences arise in the context of meditation?” Many other important questions will need to be pursued through additional theory-driven research.

This study has a number of other limitations. Demand characteristics and subject expectations are an inherent limitation of many types of studies, including nearly all intervention studies, meditation studies, and pharmacology studies. In clinical meditation studies, the simple fact that the name of a meditation program includes the phrase “stress reduction” sets up expectations that the program will reduce stress. Similarly, certain experiences may be more or less frequently reported depending on the subject’s expectations of what is supposed to happen in the context of meditation. There are several reasons why the influence of subjects’ expectations are minimized in this study. First, lights are not commonly described in American Buddhist meditation literature and are likely not well known to most American practitioners. Participants in this study were deliberately recruited on the basis that the experiences they had with meditation were *unexpected.* In our sample, only one participant made an explicit association between their own experience and Buddhist theories about the significance of lights. The subjects also tended to describe their experience in non-Buddhist terms such as the “curtain of light,” “Christmas lights,” “electrical sparks,” or “little stars.”

It may seem plausible that the light-related experiences in our sample were just hypnagogic hallucinations that were caused by falling asleep and not by meditation practice. We find it unlikely that sleep played a role in these experiences for several reasons. First, hypnagogic hallucinations are extremely common (Ohayon et al., 1996) and we would therefore expect the prevalence in our sample to be much higher and also much more well known to the average meditator. Second, Buddhist meditation practice, especially among more-experienced practitioners, is associated with an increased alertness that is neurologically distinct from and resistant to sleepiness (Britton et al., 2013). Third, as described previously, hypnagogic hallucinations are phenomenologically and neurologically different from waking hallucinations that arise in the context of sensory deprivation or perceptual isolation (Cheyne et al., 1999a,b; Ohayon, 2000; Wackermann et al., 2002; Collerton and Perry, 2011; Fenelon, 2013). Finally, several of our subjects reported that their eyes were open during these experiences, and mentioned them in conjunction with concentration, never drowsiness or sleep. Nevertheless, future studies of meditation-induced light experiences should include real-time measurements of brain activity to rule out this possibility.

Now that some of the basic phenomenology of meditation-related light experiences has been described, we can begin to investigate follow-up questions in a hypothesis-driven experimental design that uses quantitative statistical analyses. Future studies would benefit from investigating light-related experiences in larger sample of practitioners, with a range of practice types and durations, including secular, clinical meditation practices (e.g., MBSR). The hypothesized link between concentration and meditation-induced light experiences could be empirically investigated with neuropsychological tests of attention (e.g., SART) and concurrent neuroimaging (fMRI, EEG). Real-time neuroimaging concurrent with reports of light experiences may be able to determine neurological mechanisms as well as the possible relationship to hypnagogic hallucinations. Future studies should also make an effort to control for light exposure, both in terms of the ambient light in the environment, and in terms of whether the eyes are open or closed. Changes in light exposure can result in compensatory changes in the retina (dark adaptation) that may also cause short-lived changes in visual experience (Lamb and Pugh, 2004). Similarly, visual after-effects are also brief adaptations in response to certain stimuli that may be mediated at the level of the eye or brain (Rhodes et al., 2003). Because eye-based changes are less enduring than brain-based changes, more information about the duration of meditation-induced light experiences may also help elucidate their underlying sensory and neural mechanisms.

## CONCLUSION

This paper demonstrates importance of engaging with traditional Buddhist presentations of the states and stages of meditation, as what is described in the texts in many cases is very closely linked with reports of meditation experience derived from contemporary practitioners. By investigating traditional Buddhist sources, meditation researchers and clinicians will be more informed about the varieties of meditation experiences and their possible significance. Investigating meditation-induced light experiences suggests that on account of restricting attention by deselecting sensory stimuli, certain meditation practices may function in a manner analogous to sensory deprivation and perceptual isolation. The arising of lights may signal a period of enhanced neuroplasticity and potential for important and enduring shifts. Further research should investigate whether it is the unique configuration of sensory deprivation, attentional training, and investigative processes that accounts for why meditative practices tend to lead to enduring perceptual and affective changes and cognitive insights.

## Conflict of Interest Statement

The authors declare that the research was conducted in the absence of any commercial or financial relationships that could be construed as a potential conflict of interest.

## Footnotes

1 The total percentage surpasses 100% due to some participants practicing in multiple traditions.
